# HIV-1 Rev-RRE functional activity in primary isolates is highly dependent on minimal context-dependent changes in Rev

**DOI:** 10.1038/s41598-022-21714-2

**Published:** 2022-11-01

**Authors:** Godfrey Dzhivhuho, Jordan Holsey, Ethan Honeycutt, Heather O’Farrell, David Rekosh, Marie-Louise Hammarskjold, Patrick E. H. Jackson

**Affiliations:** 1 Myles H. Thaler Center for HIV and Human Retrovirus Research, University of Virginia, Charlottesville, VA USA; 2 Department of Microbiology, Immunology, and Cancer Biology, School of Medicine, University of Virginia, Charlottesville, VA USA; 3 Division of Infectious Diseases and International Health, School of Medicine, University of Virginia, Charlottesville, VA USA

**Keywords:** Microbiology, Virology, Viral pathogenesis

## Abstract

During HIV infection, intron-containing viral mRNAs are exported from the cell nucleus to the cytoplasm to complete the replication cycle. Cellular restrictions on the export of incompletely spliced transcripts are overcome by a viral protein, Rev, and an RNA structure found in all unspliced and incompletely spliced viral mRNAs, the Rev Response Element (RRE). Primary HIV isolates display substantial variation in the sequence and functional activity of Rev proteins. We analyzed Rev from two primary isolates with disparate activity that resulted in differences in in vitro fitness of replication-competent viral constructs. The results showed that amino acid differences within the oligomerization domain, but not the arginine-rich motif or the nuclear export signal, determined the level of Rev activity. Two specific amino acid substitutions were sufficient to alter the low-activity Rev to a high-activity phenotype. Other mutations in Rev sequences had unpredictable effects on activity that differed between the two Rev backbones. The sensitivity of Rev function level to small sequence changes likely permits modulation of Rev-RRE activity during HIV infection, which may play a role in pathogenesis. The functional consequences of Rev mutations differed between primary isolates, highlighting the challenge of generalizing studies of Rev conducted using laboratory HIV strains.

## Introduction

Retroviruses must export intron-containing viral mRNAs from the cell nucleus to the cytoplasm in order to complete their replication cycles. So-called “complex” retroviruses utilize a *trans*-acting protein and a *cis-*acting RNA secondary structure to overcome the cellular restriction on the export of unspliced and incompletely spliced mRNAs^1^. The complex retrovirus HIV-1 requires both the viral protein Rev and an RNA structure termed the Rev-Response Element (RRE) to accomplish this process^2,3^. During HIV replication, Rev is translated from a completely spliced, and thus constitutively exported, mRNA species^4–6^. After translation, Rev is imported into the nucleus where it binds to the RRE found on all unspliced and incompletely spliced viral mRNAs^7–10^. A Rev homo-oligomer forms that recruits cellular factors including Crm1 and Ran-GTP. The resulting ribonucleoprotein complex is then exported to the cytoplasm where the intron-containing viral mRNA is translated or packaged into progeny viruses^11–13^.

Primary isolates of HIV exhibit sequence variation throughout the genome, including in the regions encoding *rev* and the RRE^14^. Variations in both *rev* and the RRE are observed not only between primary isolates sequenced from different hosts, but also within viral quasispecies arising during the course of infection in a single host. Sequence changes in *rev*, the RRE, or both of these elements can give rise to differences in the functional activity of the Rev-RRE regulatory axis^15–18^.

**Figure 1:**
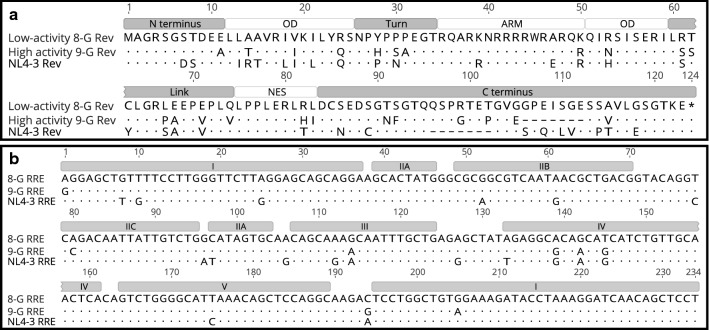
Alignment of Rev and RREs. (**a**) The amino acid sequences of the Revs corresponding to the 8-G, 9-G, and NL4-3 viruses were aligned. Differences relative to the 8-G sequence are highlighted. Dots represent identical amino acid residues to the 8-G sequence, while dashes represent a deletion relative to 8-G. Functional domains are marked above the alignment. (**b**) The nucleic acid sequences of the 234-nt RREs corresponding to the 8-G, 9-G, and NL4-3 viruses were aligned. Differences relative to the 8-G sequence are highlighted. Dots represent an identical base to 8-G. Regions that would fold to form a five stem-loop RNA structure are noted above the sequence. OD—oligomerization domain, ARM—arginine rich motif, NES—nuclear export signal.

The Rev gene in subtype B laboratory isolates, such as NL4-3, that have been used in most studies to date encodes a 116 amino acid protein (Fig. 1a). Rev contains several functional domains, separated by less ordered spacer regions. The core functional domains are the bipartite oligomerization domain (OD), the arginine-rich motif (ARM), and the nuclear export signal (NES). The OD consists of two alpha-helical regions which stabilize the Rev monomer and permit the formation of homodimers and higher order Rev oligomers^19^. The ARM is the site of direct interaction with the RRE and doubles as a nuclear localization signal^20–23^. The NES permits Rev oligomer interaction with Crm1 to accomplish nuclear export^3,24^. The N-terminal and C-terminal regions of Rev are less well ordered. A “Turn” region is located between the first portion of the OD and the ARM, and a “Link” region is located between the second portion of the OD and the NES^25^.

We previously described twelve HIV-1 primary isolates that displayed substantial variation in the activity of the Rev-RRE regulatory axes^15^. Within this set, we found two subtype G viruses with widely disparate Rev-RRE activity due to differences between the Rev proteins (Fig. 1a) rather than the RREs (Fig. 1b). Furthermore, more of the lower-activity subtype G Rev protein was present at steady state than the high-activity Rev, suggesting that the activity difference was not attributable to the amount of protein produced from these sequences.

Previous work describing differential Rev activity in primary isolates has been restricted to studies of subtype B and C viruses. Rev and RRE variation in other HIV subtypes has not been well characterized. In this study, we sought to identify the key sequence determinants of Rev functional activity, using the two subtype G primary isolates as a model.

## Results

### Determination of native Rev functional activity

We previously described the relative functional activity of the two subtype G Revs using a transient transfection-based lentiviral vector assay system (described in^18^). In that system, vector RNA was packaged in a Rev-RRE dependent fashion and the resulting vector titer corresponded to the relative activity level of the Rev-RRE pair. Using this assay, the 8-G Rev/NL4-3 RRE pair was found to have notably low functional activity, while the 9-G Rev/NL4-3 RRE pair had high functional activity^15^. The finding of differential Rev activity was consistent when the two Revs were paired with the RRE from either the 8-G or 9-G primary isolate as well.

**Figure 2:**
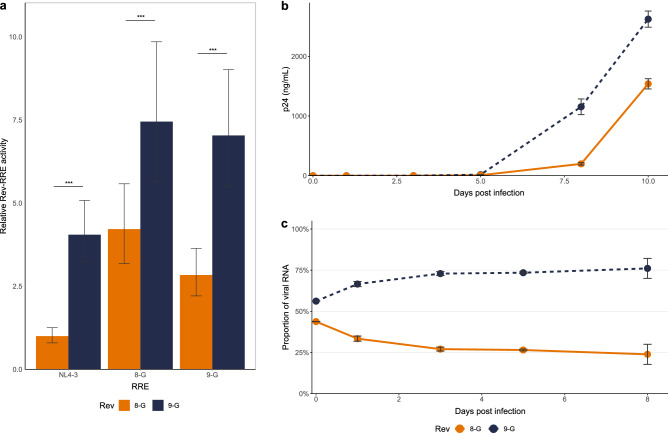
Relative activity of the 8-G and 9-G Revs. The activity of the Revs derived from the 8-G and 9-G primary isolates was determined via three complementary methods. (**a**) Rev-RRE activity was determined by a fluorescence-based assay utilizing lymphoid cells transduced with Rev- and RRE-containing reporter constructs. The relative activity of the 8-G and 9-G Revs was measured in conjunction with the NL4-3, 8-G, and 9-G RREs. Relative activity is expressed in arbitrary units with the activity of the 8-G Rev/NL4-3 RRE pair defined as 1. *N* > 3 for all comparisons, error bars represent 95% confidence intervals (CI), ***p < 0.001. (**b**) Replication-competent constructs containing the 8-G or 9-G Rev were used to infect parallel cultures of SupT1 cells. Infection kinetics were measured by determining the production of p24 protein in culture supernatants over time. *N* = 3 for each data point, error bars represent standard deviation (SD). (**c**) The relative replicative fitness of the replication-competent constructs assayed in (**b**) was also measured in a direct competition assay. Cultures of SupT1 cells were co-infected with 8-G and 9-G Rev containing viruses. The relative amount of 8-G Rev and 9-G Rev sequence in viral cDNA was determined at multiple time points, and expressed as the percentage of total Rev cDNA at that time point. *N* = 3 for each data point, error bars represent SD.

To validate these results, a second assay system for determining Rev-RRE functional activity was used that better reproduces the natural process of viral infection. This fluorescence-based system measures activity from integrated proviruses in lymphoid cells, as opposed to transiently transfected plasmid DNA. This assay was described fully in^26^. Using this system, the relative functional activity of 8-G Rev was again found to be significantly lower than that of the 9-G Rev, as measured in combination with the NL4-3 RRE (*p* < 0.001) (Fig. 2a). This difference in activity persisted when the Revs were tested in combination with RREs from the 8-G and 9-G primary isolates (*p* < 0.001 for both comparisons).

To confirm this finding, the activities of the two Revs were also analyzed using a spreading infection assay. The laboratory HIV strain NL4-3 was modified with two stop codons in the first coding exon of Rev (without altering the Tat sequence) to silence native *rev* expression, and a *rev*-*nef* cassette was inserted in the *nef* position with the two genes separated by an internal ribosomal entry site (IRES). This resulted in a replication-competent construct expressing all viral genes with an exchangeable *rev* in a heterotopic position*.* Viruses were created containing the 8-G or the 9-G *rev* and used to infect parallel cultures of SupT1 cells. Viral growth kinetics were determined by serial measurements of p24 starting on the day of infection. The virus containing 9-G *rev* yielded a higher amount of p24 at an earlier time after infection than did the virus containing 8-G *rev* (Fig. 2b). This difference in the p24 production curves is consistent with a significant difference in replication kinetics^27^.

Finally, the difference in viral replication due to the different *rev* sequences was verified by a direct competition assay. Cultures of SupT1 cells were co-infected with the 8-G and 9-G *rev*-containing viruses. After infection, a portion of the culture supernatant was collected at serial time points and viral mRNA was prepared. The relative amount of 8-G and 9-G viral RNA at each time point was determined by PCR. The ratio of 9-G construct RNA to 8-G construct RNA increased over time, consistent with greater fitness of the 9-G *rev* containing virus (Fig. 2c).

### Creation of chimeric Rev constructs

The 8-G and 9-G Rev sequences differ from each other at 29 amino acid positions, with substitutions in all functional regions and a seven amino acid deletion in the 9-G C-terminal region relative to 8-G (Fig. 1a). Chimeric *rev* sequences were designed for use in the fluorescence-based functional assay consisting of either the 8-G or 9-G sequence with one or more functional domains swapped for the sequence corresponding to the other Rev. For example, the “8-G with 9-G Turn” *rev* was identical to 8-G except for the Turn region (aa 26 to 34), the sequence of which was identical to the corresponding region from 9-G. Additional sequences were designed with single or multiple amino acid substitutions at key positions. Designed *rev* sequences were cloned into packageable constructs for the performance of functional assays. An alignment of each Rev used in these studies can be found in the Supplemental Material (Fig. S1).

### Identification of Rev functional domains responsible for differential activity

**Figure 3:**
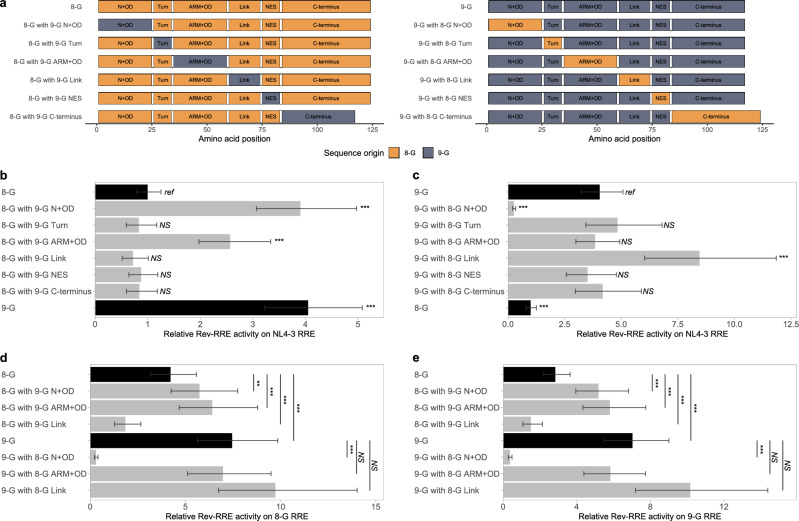
Activity of chimeric Rev sequences. Chimeras of 8-G and 9-G Rev were created by exchanging portions of the sequence corresponding to specific functional domains. A schematic of each Rev construct used in this set of experiments is shown in (**a**). For example, the “8-G with 9-G N + OD” chimera consists of sequence from 8-G Rev except for the functional domains of the N-terminus and first oligomerization domain (corresponding to amino acid positions 1–25) which is taken from 9-G Rev. Functional activity was determined using the fluorescence-based assay system. In each plot, the native 8-G and 9-G Rev sequences are included in dark bars for reference and chimeras are in light bars. (**b**) 8-G chimeras tested on the NL4-3 RRE. (**c**) 9-G chimeras tested on the NL4-3 RRE. (**d**) Selected chimeras tested on the 8-G RRE. (**e**) Selected chimeras tested on the 9-G RRE. For plots b and c, the statistical comparison is performed in reference to the top-most Rev, while in plots d and e the two Revs being compared are indicated by the vertical bar. For all plots, relative activity is expressed in arbitrary units with the activity of the 8-G Rev/NL4-3 RRE pair defined as 1. *N* ≥ 3 for all data points, error bars represent 95% CI. N + OD—N-terminus plus first portion of the oligomerization domain, ARM + OD—arginine rich motif plus second portion of the oligomerization domain, NES—nuclear export signal. *Ref*—reference sequence for the plot, *NS*—not significant, ***p < 0.001, **p < 0.01.

To try to define the regions of Rev that determined the difference in activity, chimeric *rev* sequences were tested on three RREs in our functional activity assay (Fig. 3a). Chimeras based on 8-G *rev* were first tested with the NL4-3 RRE in our functional activity assay (Fig. 3b). Two 8-G-based *rev* chimeras displayed increased activity relative to the native 8-G sequence: one with a 9-G *rev* sequence at the N-terminus and the first portion of the OD (8-G with 9-G N + OD (Fig. S1 #2)) and a second one with a 9-G *rev* sequence at the ARM and second portion of the OD (8-G with 9-G ARM + OD (Fig. S1 #4)) (*p* < 0.001 for both comparisons). The 8-G *rev* chimeras with substituted 9-G Turn (Fig. S1 #3), Link (Fig. S1 #5), or NES (Fig. S1 #6) regions did not significantly differ in activity from the native 8-G *rev*.

Complementary chimeras based on the 9-G *rev* sequence were also tested with the NL4-3 RRE (Fig. 3c). Replacement of the 9-G N-terminus and first portion of the OD region with sequence from 8-G (9-G with 8-G N + OD (Fig. S1 #27)) led to significantly lower functional activity than native 9-G *rev* sequence (*p* < 0.001), while replacement of the Link region (Fig. S1 #30) resulted in greater functional activity (*p* < 0.001). In contrast to the 8-G-based chimera, substitution of the ARM and second portion of the OD (Fig. S1 #29) did not alter functional activity in the 9-G backbone context (*p* = 0.89).

Functional activities of the 8-G and 9-G *rev* chimeras were also tested with the 8-G RRE (Fig. 3d) and 9-G RRE (Fig. 3e). On these RREs, both the 8-G with 9-G N + OD *rev* and the 8-G with 9-G ARM + OD *rev* chimeras consistently displayed higher functional activity than native 8-G Rev (*p* < 0.001 for all comparisons). For the remaining *rev* chimeras, the pattern of activity alteration was similar across all three RREs.

Rev constructs with modifications of the C-terminal region were also tested on each of the three RREs (Fig. S2a). Exchange of the C-terminal region did not significantly affect functional activity of either the 8-G or 9-G *rev* sequence on any of the three RREs (Fig. 3b, c, Fig. S2b–d) (Fig. S1 #7 and 32). Replicating the seven amino acid insertion/deletion within the C-terminus also had no effect on activity (Fig. S1 #25 and 50), while truncation of the C-terminus after position 83 in both Revs (Fig. S1 #24 and 49) significantly decreased functional activity relative to the corresponding native sequence (*p* < 0.001) (Fig. S2b–d).

### Functional activity alteration as assessed by single amino acid substitutions

**Figure 4:**
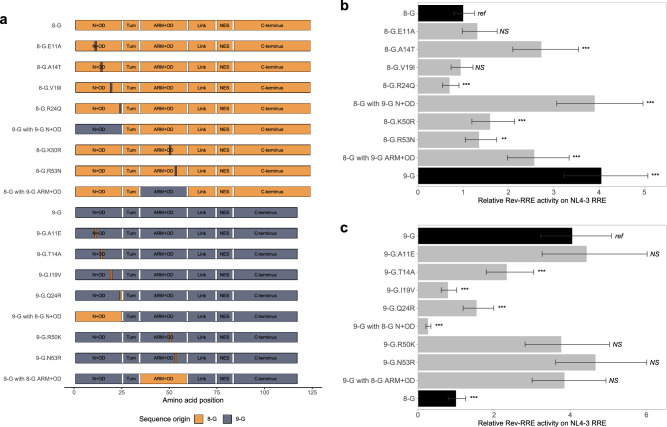
Activity of Revs with single amino acid substitutions on NL4-3 RRE. 8-G and 9-G Revs with single amino acid substitutions within the N-terminal, OD, or ARM regions were created. A schematic of each Rev used in this set of experiments is shown in (**a**). Single amino acid substitutions are shown as a vertical bar on contrasting color at the indicated location. The functional activity of the 8-G (**b**) and 9-G (**c**) constructs was determined on the NL4-3 RRE using the fluorescence-based assay system. In each plot, the native 8-G and 9-G Rev sequences are included in dark bars for reference and modified Revs are in light bars. For both plots, relative activity is expressed in arbitrary units with the activity of the 8-G Rev/NL4-3 RRE defined as 1. The statistical comparison is performed in reference to the top-most Rev in each plot. *N* ≥ 3 for all data points, error bars represent 95% CI. N + OD—N-terminus plus first portion of the oligomerization domain, ARM + OD—arginine rich motif plus second portion of the oligomerization domain, NES – nuclear export signal. *Ref*—reference sequence for the plot, *NS*—not significant, ***p < 0.001, **p < 0.01.

Having identified substitutions within the N-terminal, OD, and ARM domains as key for defining Rev functional activity, mutants with single amino acid changes within these regions were created (Fig. 4a).

Four amino acid differences between the native 8-G and 9-G Rev sequences occurred in the N-terminus and first portion of the OD, at positions 11, 14, 19, and 24. Only the 8-G.A14T (Fig. S1 #12) mutant displayed significantly greater activity than the native 8-G Rev on the NL4-3 RRE (*p* < 0.001) (Fig. 4b). The 8-G.E11A (Fig. S1 #8) and 8-G.V19I (Fig. S1 #17) mutants displayed similar activity to 8-G Rev, while the 8-G.R24Q (Fig. S1 #21) mutant displayed significantly lower activity (*p* = 0.001). Reciprocal substitutions in the 9-G Rev context showed a significant decrease in activity for the 9-G.T14A (Fig. S1 #34), 9-G.I19V (Fig. S1 #43), and 9-G.Q24R (Fig. S1 #46) mutants relative to native 9-G Rev, while the 9-G.A11E (Fig. S1 #33) substitution did not change activity (Fig. 4c).

Two amino acid differences occurred within the ARM and second portion of the OD at positions 50 and 53. Both the 8-G.K50R (Fig. S1 #22) and the 8-G.R53N (Fig. S1 #23) substitutions increased functional activity over native 8-G Rev on the NL4-3 RRE (*p* < 0.01) (Fig. 4b). The corresponding substitutions 9-G.R50K (Fig. S1 #47) and 9-G.N53R (Fig. S1 #48) did not alter activity relative to native 9-G Rev (Fig. 4c).

A similar pattern in activity level was seen when the Rev mutants were tested on the 8-G and 9-G RREs (Fig. S3). The 8-G.A14T Rev displayed consistently greater activity than the native sequence, while 8-G.R24Q displayed consistently lower activity (*p* ≤ 0.01 for all comparisons).

### Functional activity alteration with multiple amino acid substitutions

**Figure 5:**
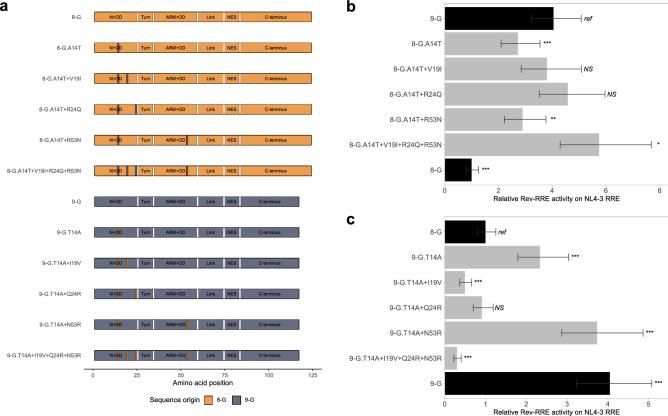
Activity of Revs with multiple amino acid substitutions on NL4-3 RRE. 8-G and 9-G Revs with multiple amino acid substitutions were created. A schematic of each Rev used in this set of experiments is shown in (**a**). Single amino acid substitutions are shown as a vertical bar on contrasting color at the indicated location. The functional activity of 8-G (**b**) and 9-G (**c**) constructs on the NL4-3 RRE using the fluorescence-based assay system is shown. In each plot, the native 8-G and 9-G Rev sequences are included in dark bars for reference and modified Revs are in light bars. For both plots, relative activity is expressed in arbitrary units with the activity of the 8-G Rev/NL4-3 RRE pair defined as 1. The statistical comparison is performed in reference to the top-most Rev in each plot. *N* ≥ 3 for all data points, error bars represent 95% CI. N + OD—N-terminus plus first portion of the oligomerization domain, ARM + OD – arginine rich motif plus second portion of the oligomerization domain, NES—nuclear export signal. *Ref*—reference sequence for the plot, *NS*—not significant, ***p < 0.001, **p < 0.01, *p < 0.05.

Rev mutants incorporating combinations of amino acid substitutions were created to identify the minimum change required to define the activity phenotype (Fig. 5a). While the 8-G.A14T substitution was sufficient to significantly increase the activity of 8-G Rev over the native sequence, the mutant was still less active than the native 9-G Rev (*p* < 0.001). The 8-G mutants 8-G.A14T + V19I (Fig. S1 #13) and 8-G.A14T + R24Q (Fig. S1 #14), however, displayed functional activity that was similar to 9-G on the NL4-3 RRE (Fig. 5b). This pattern of activity was replicated with the 8-G and 9-G RREs (Fig. S4a and c).

9-G Rev mutants containing the corresponding amino acid substitutions were also created and tested. On the NL4-3 RRE, the mutants 9-G.T14A + Q24R (Fig. S1 #39) and 9-G.T14A + I19V (Fig. S1 #38) displayed similar or lower activity than the native 8-G Rev (Fig. 5c). This pattern held for the 8-G and 9-G RREs as well (Fig. S4b and d).

### Effect of phosphomimetic substitutions

The amino acid residue at position 14 was found to be a key determinant of activity. One potential explanation for the increase in 8-G activity with the A14T substitution (and corresponding decrease in 9-G activity with the T14A substitution) could be linked to the potential for threonine, but not alanine, to become phosphorylated. The amino terminus of Rev is phosphorylated *in vivo*^28,29^, and Rev phosphorylation at other sites (but not position 14) has been associated with differences in binding to the RRE^30^. To test whether phosphorylation of 14 T could affect activity, phosphomimetic substitution mutants were created at this position^31^. A 14S substitution presents an alternative phosphorylation target, while 14D and 14E substitutions structurally mimic phosphothreonine. The 14A substitution constitutes a site without potential for phosphorylation.

**Figure 6:**
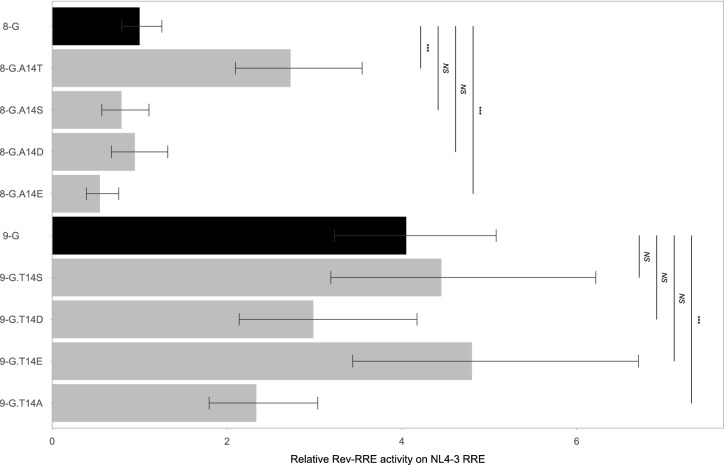
Activity of Revs with phosphomimetic substitutions on NL4-3 RRE. Revs with phosphomimetic amino acid substitutions at position 14 were created and their functional activity determined on the NL4-3 RRE using the fluorescence-based assay system. The native 8-G and 9-G Rev sequences are included in dark bars for reference and modified Revs are in light bars. Relative activity is expressed in arbitrary units with the activity of the 8-G Rev/NL4-3 RRE pair defined as 1. The statistical comparison is performed is performed between the Revs indicated by the vertical bars. *N* ≥ 3 for all data points, error bars represent 95% CI. *NS*—not significant, ***p < 0.001.

On the NL4-3 RRE, the phosphomimetic 8-G.A14S (Fig. S1 #11), 8-G.A14D (Fig. S1 #9), and 8-G.A14E (Fig. S1 #10) mutants displayed similar or lower activity to the native 8-G sequence, and only the 8-G.A14T mutant showed higher activity (Fig. 6). In the 9-G context, the 9-G.T14S (Fig. S1 #37), 9-G.T14D (Fig. S1 #35), and 9-G.T14E (Fig. S1 #36) mutations did not significantly change 9-G activity and only 9-G.T14A mutant activity differed from the native sequence. A similar pattern of activity was seen on the 8-G and 9-G RREs (Fig. S5). Thus, while the phosphomimetic substitutions in the 9-G Rev context preserved a high level of activity, none of the corresponding substitutions in the 8-G Rev context increased activity similarly to that displayed by 8-G.A14T. Phosphorylation of position 14 is thus unlikely to explain the observed differences in functional activity.

### Structural predictions of 8-G and 9-G Rev

**Figure 7:**
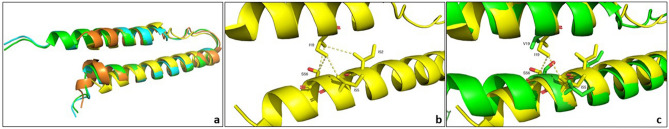
Rev structures. 8-G and 9-G Rev structures were predicted using AlphaFold Colab and compared to published crystal structures of NL4-3 Rev with (PDB: 4PMI) and without (PDB: 3LPH) RNA. The 8-G and 9-G proteins were truncated at residue 68, to correspond with the NL4-3 structures; the remainder of the protein chains were disordered in the predicted structures, with little to no secondary structure. (**a**) Overlay of the four structures. Unbound NL4-3 Rev is shown in orange, RNA-bound NL4-3 in yellow, 8-G in green, and 9-G in cyan. (**b**) The OD helices of RNA-bound NL4-3 Rev, showing the interaction of I19 with I52, I55 and S56. The highlighted residues are conserved in the 9-G sequence. c. The same protein region as in b, with the 8-G Rev overlaid in green.

Predicted structures of the native 8-G and 9-G Revs were computed using AlphaFold Colab^32^ and visualized using PyMOL Molecular Graphics System, version 2.5.1 (Schrödinger, LLC). The resulting predictions were compared to the NL4-3 Rev dimer crystal structures previously published by Frankel and coworkers^33,34^. The predicted subtype G structures overlapped each other and both of the NL4-3 crystal structures (Fig. 7a). Isoleucine 19, conserved in the NL4-3 Rev and 9-G Rev sequences, is positioned between the two OD helices within the Rev monomer. In the RNA-bound crystal structure this residue is expected to interact with the I52, I55, and S56 residues found in all three Revs (Fig. 7b). However, in the 8-G Rev sequence, position 19 is a valine, which may not be able to form the monomer-stabilizing interactions with residues I55 and S56 (Fig. 7c). We hypothesized that this difference could account for the decreased activity of the 9-G.I19V mutant relative to native 9-G as well as the increased activity seen with the 8-G.A14T + V19I mutant relative to 8-G.A14T.

**Figure 8:**
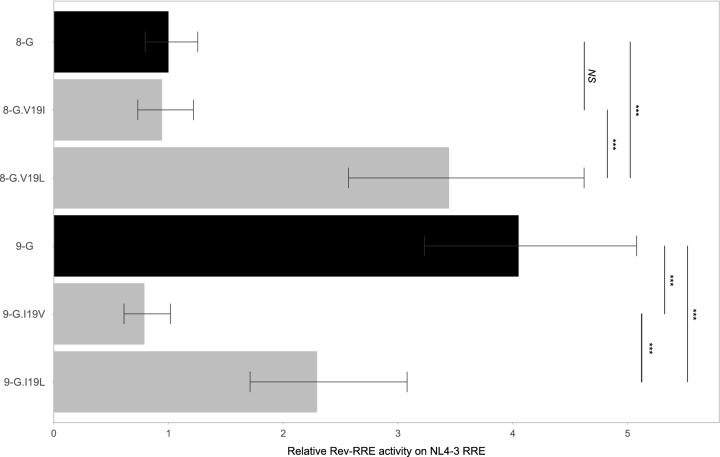
Activity of position 19 mutants on NL4-3 RRE. Revs with amino acid substitutions at position 19 were created and their functional activity determined on the NL4-3 RRE using the fluorescence-based assay system. The native 8-G and 9-G Rev sequences are included in dark bars for reference and modified Revs are in light bars. Relative activity is expressed in arbitrary units with the activity of the 8-G Rev/NL4-3 RRE pair defined as 1. The statistical comparison is performed between the Revs indicated by the vertical bars. *N* ≥ 3 for all data points, error bars represent 95% CI. *NS*—not significant, ***p < 0.001.

To test this hypothesis, 8-G and 9-G mutants with a 19L substitution were generated and tested on the NL4-3 RRE (Fig. 8). While the 8-G.V19I (Fig. S1 #17) substitution alone did not alter activity relative to native 8-G, the 8-G.V19L (Fig. S1 #18) mutant displayed significantly greater activity (*p* < 0.001). In the 9-G context, both 9-G.I19V (Fig. S1 #43) and 9-G.I19L (Fig. S1 #42) displayed lower activity than the native 9-G sequence (*p* < 0.001 for both comparisons), but 9-G.I19L displayed greater activity than 9-G.I19V (*p* < 0.001). A similar pattern of activity was seen with the 8-G and 9-G RREs (Fig. S6. Thus, for both Revs the 19L variants displayed consistently greater activity than the 19 V variants. However, the 19I variant yielded low activity in the 8-G context but high activity in the 9-G context. Thus the role of the residue at position 19 in stabilizing the Rev monomer may not explain the activity variation.

## Discussion

This report adds to the sparse literature on the functional implications of naturally occurring variation in the HIV-1 Rev-RRE regulatory axis. To our knowledge, this is the first functional exploration of Rev variation in subtype G primary isolates. Using two Rev sequences with markedly different levels of activity, we demonstrated that amino acid differences within the oligomerization domain alone result in significant variation in Rev activity. Furthermore, just two amino acid substitutions in this region are sufficient to alter a low-activity to a high-activity Rev phenotype.

Modulation of the activity of the Rev-RRE axis may be a mechanism by which HIV adapts to an array of selection pressures that the virus encounters throughout the course of natural infection. The virus may replicate in the environment of the genital or rectal mucosa during transmission, in various anatomical compartments including blood and lymphoid tissue during chronic infection, and in conditions of attenuated immune surveillance during late disease. Experimental evidence suggests that low Rev-RRE functional activity is associated with evasion of T-cell mediated killing^35^, while high RRE activity may result in a more rapid decline in CD4 count^36,37^. The ability to modulate replication kinetics and the presentation of viral antigens through differences in Rev-RRE functional activity may offer an advantage to viruses in differing fitness landscapes.

Previous analysis of Rev variation in primary isolates has been limited to subtype B^38^ and C^39^ viruses. In these subtypes, changes in the Rev oligomerization domain^40,41^, NES^42,43^, and C-terminal region^44^ have been associated with differences in activity. In a subtype B context, mutations at amino acid position 18 within the first OD were found to significantly alter Rev-dependent reporter gene expression independent of changes in oligomerization on the RRE^40^. Our results in subtype G isolates are consistent with the hypothesis that the Rev OD is important for activity modulation.

The specific amino acid substitutions that significantly affect Rev activity in these subtype G sequences, at positions 14 and 19, have not been previously described as being functionally important. Fernandes and coworkers performed an analysis of Rev functional activity using the subtype B NL4-3 virus as a model^45^. Each amino acid position in Rev was mutated, and the relative replicative fitness of viruses with Rev mutations was determined. In this model, an R24Q substitution resulted in a substantial increase in fitness; E11A and A14T resulted in a slight increase in fitness; V19I and R53N had no substantial change; and K50R showed a substantial decrease in fitness. These single amino acid substitutions had very different effects in the context of the 8-G sequence. For example, while our assay system replicates the finding from Fernandes that glutamine (Q) rather than arginine (R) at position 24 yields significantly higher NL4-3 Rev activity (data not shown), in 8-G, R24Q displayed significantly lower activity than the corresponding native Rev. On the other hand, the mutation Q24R in the 9-G Rev context yields lower activity, consistent with the finding of Fernandes and coworkers for the NL4-3 Rev.

Because the functional impact of individual amino acid substitutions is highly context-dependent, making predictions of function based on structure is challenging. The NL4-3 Rev crystal structure suggests that the residue at position 19 may be functionally important due to a role in stabilizing the interaction of the two OD regions within the Rev monomer. While this hypothesis can explain alterations in the activity of the 9-G Rev, in which both isoleucine and leucine at position 19 yield higher activity than valine, it does not predict activity in the 8-G context, where isoleucine and valine at position 19 showed similar low levels of activity. Thus, while structural studies of Rev utilizing NL4-3 or other laboratory strains of HIV can yield valuable insights, the functional significance of individual amino acid substitutions cannot be generalized between even closely related viruses. Mechanistic investigations of HIV must take a broader view of viral diversity and test hypotheses in primary isolates as well as laboratory strains.

A corollary of the highly context-dependent consequence of single amino acid substitutions is that combinations of substitutions have unpredictable implications for Rev functional activity. The effect of multiple substitutions is not merely additive. While 8-G.V19I had similar activity to native 8-G, the addition of substitution V19I to A14T increased 8-G activity more than the A14T substitution alone.

Our results suggest that Rev functional activity is highly sensitive to small sequence changes. We previously described HIV-1 primary isolates with variable Rev-RRE functional activity attributable to differences in Rev, the RRE, or both^15^. Single nucleotide substitutions within the RRE can give rise to different two-dimensional structures that, in turn, have significant differences in functional activity^17^. As the RRE is located within a region of the HIV genome that codes only for *env*, we considered that there might be a lower barrier for modulation of Rev-RRE functional activity through changes in the RRE, as this can be accomplished with a minimal number of *env*-synonymous mutations. On the other hand, *rev* overlaps with *tat, env,* or both over its entire length, which might be expected to constrain *rev* mutations that could modulate Rev-RRE activity. Our study now demonstrates that, in HIV-1 primary isolates, one or two amino acid substitutions within the oligomerization domain of Rev is sufficient to cause substantial alteration in activity. Given this substantial flexibility to alter Rev activity with minimal sequence changes, we would expect variations in both *rev* and the RRE to have the capacity to modulate Rev-RRE activity during an HIV infection, without a clear preference for RRE mutations. The Frankel group has observed that the potential for *rev* sequence variation is further enhanced by the fact that the functional regions of *tat* and *rev* are spatially segregated, such that mutations within key domains of *rev* would not be expected to have significant functional implications for *tat*, and *vice versa*^45^.

In this study, we limited our attention to correlations between Rev sequence and functional activity. While we can exclude some explanations for differences in Rev activity, specifically phosphorylation at position 14 and stabilization of the Rev monomer at position 19, the mechanisms behind the observed variations in activity have not been defined. While we previously found that the steady state protein level of 8-G exceeded that of 9-G^15^, it is still possible that differences in protein steady state level or turnover kinetics, rather than intrinsic differences in Rev activity, could account for the functional activity variations of some of the mutants analyzed in these assays. The localization of functionally important amino acid substitutions within the OD does not necessarily implicate the process of Rev oligomerization in activity variation. From the standpoint of viral adaptation to selection pressures, however, it is immaterial whether differential Rev-RRE activity is achieved via any of an array of potential mechanisms. A Rev mutation that accomplishes a decrease in Rev-RRE activity through decreased Rev protein level may be equally useful to the virus as a mutation that decreases the affinity of the Rev-RRE nucleoprotein complex for Crm1. While elucidating molecular mechanisms of Rev-RRE activity variation remains an important question, it is a separate question from the relationship between Rev sequence variation and the activity of the Rev-RRE axis.

In this study, we describe novel amino acid substitutions within the oligomerization domain of Rev that give rise to significant differences in Rev-RRE functional activity, specifically in the context of subtype G Rev. We hypothesize that modulation of the Rev-RRE regulatory axis is one mechanism by which HIV can alter its replication kinetics and the relative expression of immune-activating and immune-modulatory viral proteins^35^. This would give the virus a means to adapt to the differing immune milieus that it encounters during the course of disease progression. Additional studies of this regulatory axis in primary isolates from different clinical scenarios are needed to define the potential role of the Rev-RRE system in the pathogenesis of HIV disease.

## Methods

### Rev and RRE sequence selection

The *rev* and RRE sequences for these studies were obtained from one of two primary isolates (8-G, Genbank: FJ389367^46^; 9-G, Genbank: JX140676^47^) or from the laboratory strain NL4-3 (Genbank: U26942^48^). The sequences corresponding to the *rev* or RRE from each viral genome were analyzed and extracted using Geneious Prime v11.0.9 (Biomatters).

### Replication competent constructs and viral stocks

Replication competent HIV constructs containing the 8-G and 9-G *rev* sequences were constructed to determine differences in replication kinetics. Full-length NL4-3 HIV^49^ was modified to silence native *rev* expression by mutating the initial AUG codon to ACG and changing the codon at position 23 (UAU: Y) to a stop codon (UAA) without altering the sequence of Tat. The native *nef* was replaced, beginning at the start codon, by a cassette consisting of *rev* and *nef* separated by an internal ribosomal entry site (IRES). The *rev* sequence was derived from either the 8-G or 9-G virus. Plasmid numbers are given in Table S1.

To create stocks of viral constructs, 293 T/17 cells were plated at a density of 3 × 10^6^ cells per 10 cm plate in Iscove's modified Dulbecco's media (IMDM) supplemented with 10% bovine calf serum (BCS) and gentamicin. One day after plating, the cells were transfected with 15 µg of plasmid containing one of the replication-competent constructs using the polyethylenamine (PEI) method^50^. Supernatant was collected from the transfected cells 48 h post-transfection, centrifuged briefly to remove cell debris, aliquoted, and stored at −80 °C until needed. The concentration of p24 in each transfection stock was determined by ELISA as previously described^51^.

Viruses from transfection stocks were expanded by passaging in SupT1 cells. A volume of transfection stock containing 100 ng of p24 was used to infect a culture of 5 × 10^6^ SupT1 cells suspended in serum-free RPMI (PBS) in 50 mL conical tubes. Diethylaminoethyl (DEAE)-dextran was added to each culture to a final concentration of 8 µg/mL and cultures were centrifuged at 25 °C at 380 RCF for one hour. After infection, cells were washed with PBS and resuspended in SupT1 growth medium (Roswell Park Memorial Institute 1640 (RPMI) medium supplemented with 10% fetal bovine serum and gentamicin). Infected SupT1 cultures were serially passaged for up to 33 days. Every 2–3 days, half of the cells and medium were removed from each culture. The collected material was briefly centrifuged to pellet the cells, and the cell-free medium was aliquoted and frozen at -80 °C for determination of p24 and for future experiments. After each collection, the culture volume was replenished with sterile SupT1 growth medium. Medium collected on the day of peak p24 was used to determine virus titer by TCID(50) as previously described^52^.

### Replication kinetics assay

The replication kinetics of 8-G and 9-G Rev containing viruses were assayed in parallel cultures of SupT1 cells. A total of 3 × 10^5^ SupT1 cells were infected with either 8-G or 9-G Rev containing virus at a multiplicity of infection (MOI) of 0.005. Infections were performed by spinoculation in the presence of 8 µ/mL of DEAE-dextran. After infection, the cell pellet was washed with PBS once and then the cells were suspended in 10 mL SupT1 growth medium, transferred to flasks, and placed in an incubator. On days 0, 1, 3, 5, 8, and 10 after infection, 1 mL of medium with suspended cells was removed from each flask and replaced with 1 mL of sterile SupT1 growth medium. The sample was centrifuged to pellet cells, and the cell-free medium was frozen at −80 °C. After all samples were collected, the frozen medium was thawed and the concentration of p24 was determined by ELISA. Data was collected for three independent experiments.

### Competition assay

The contribution of 8-G and 9-G Rev to relative viral fitness was determined by a competition assay. A total of 3 × 10^5^ SupT1 cells were infected with both 8-G and 9-G Rev containing virus at an MOI of 0.005 for each virus stock as above. Infections were performed in triplicate. On days 1, 3, 5, and 8 after infection, 1 mL of medium with suspended cells was removed from each flask and replaced with 1 mL of sterile SupT1 growth medium. The sample was centrifuged to pellet cells, and the cell-free medium was frozen at −80 °C. Additionally, a 1:1 ratio of 8G:9G virus mix was used as a day 0 sample to determine initial viral input.

After all time points were collected, viral RNA was extracted from supernatants including the initial virus stock input. Supernatants were centrifuged at 5300 RCF for 10 min at 4 °C to remove debris, and then supernatant was transferred to a new tube. Viruses were pelleted by centrifugation at 16,000 RCF for 1 h at 4 °C and the supernatant was removed and discarded. The virus pellet was resuspended in 50 µL RNAse free 5 mM Tris–HCL, pH 8.0. The suspended viruses were treated with 10 µL proteinase K (20 mg/mL) and incubated at 55 °C for 30 min. Next, 200 µL 6 M guanidinium isothiocyanate and 10 µL glycogen (20 mg/mL) were added to each tube and samples were incubated at 25 °C for 5 min. RNA was pelleted by adding 270 µL isopropanol to each tube and centrifuging the samples at 2700 RCF for 20 min at 25 °C. The supernatant was removed, the pellet was washed with 70% ethanol, and then the RNA was resuspended in 40 µL RNAse free Tris–HCL. RT-PCR was performed using the SuperScript III Reverse Transcriptase kit (ThermoFisher) with oligo(dT)20 primer.

The relative proportion of 8-G and 9-G viral RNA was determined by PCR amplification of 2 µL of the prepared cDNA. PCR products from 8-G and 9-G containing viruses were discriminated by size on an agarose gel. PCR was performed using Taq DNA polymerase (Thermo Scientific) per manufacturer recommendations. Each reaction mixture included 2 pmol of a single forward primer located within *env* (5’-CCTAGAAGAATAAGACAGGGC-3’) and 1 pmol each of two reverse primers located within the heterotopic *rev* (8-G specific, 5’-CCCCAGATATTTCAGGCCCTC-3’; 9-G specific, 5’-GTCTCTCAAGCGGTGGTAGCAC-3’). Initial denaturation was performed at 95 °C for 3 min; then the reactions were cycled at 94 °C for 30 s, 60 °C for 30 s, and 75 °C for 45 s for 25 cycles; followed by a final extension at 72 °C for 10 min. Mixtures of known molar ratios of plasmids containing 8-G and 9-G Rev sequences were used for positive controls.

For each cDNA sample and plasmid standard, 15 uL of PCR product was loaded into each well of a 2% agarose gel containing ethidium bromide and run at 80 V for 2 h. Gels were imaged using a ChemiDoc Imager (Biorad) with exposure conditions customized to ensure that no bands were over-saturated. Bands corresponding to 8-G and 9-G Rev targets could be differentiated by size. Gel images were analyzed using Image Lab v 6.1 (Biorad) to quantify the signal from each band.

Lanes corresponding to the known plasmid mixtures were used to construct a standard curve to correlate relative band intensity to molar ratio of 8-G and 9-G Rev target sequence. Data analysis was performed in Excel 2016 (Microsoft). Relative proportion of 8-G and 9-G Rev cDNA was expressed as a percentage of the total signal from both PCR bands in each gel lane.

### Chimeric Rev constructs

Custom *rev* sequences were designed using either the 8-G or 9-G *rev* sequence. Functional regions of Rev were defined as in Fig. 1a. To create chimeras, the named functional region in either the 8-G or 9-G *rev* RNA sequence was exchanged with the RNA sequence of the corresponding region from 9-G or 8-G, respectively. For example, the “8-G with 9-G ARM + OD” sequence was identical to the 8-G *rev* sequence except from amino acid positions 35–59 in which region the corresponding sequence from 9-G *rev* was substituted. At each codon, the nucleotide sequence was taken from either 8-G or 9-G *rev*. Chimeric sequences and sequences with the specified amino acid substitutions were generated using Geneious Prime v11.0.9 (Biomatters). The amino acid sequence of each modified Rev is shown in Fig. S1 and the plasmid numbers are shown in Table S2.

### Fluorescence-based functional assays

The fluorescence-based assay of Rev-RRE functional activity, including additional details of the assay constructs, has been previously described^26^. The assay system consists of two packageable viral constructs that can be used to co-transduce target lymphoid cells and generate a fluorescent signal in a Rev-RRE dependent fashion.

The Rev-containing vector was derived from a murine stem cell virus construct (pMSCV-IRES-Blue FP), a gift from Dario Vignali (Addgene plasmid #52115). The various *rev* sequences were inserted into this construct upstream of the IRES and fluorescent marker *eBFP2* to permit co-expression from a bicistronic transcript.

The RRE-containing vector was derived from a full-length NL4-3 HIV sequence. The vector was modified in the following ways: native *rev, vpr,* and *env* expression was silenced; and the *gag* myristoylation site was deleted; *gag* was truncated and a cassette including a *cis*-acting hydrolase element from porcine teschovirus-1^53^ and *eGFP*^54^ was inserted in-frame; *nef* was deleted and replaced with *mCherry*^55^. Additionally, a 350-nt region within *env* containing the RRE was flanked by restriction sites for easy exchange, and assay constructs were created by replacing this region with the 234-nt RRE from the 8-G, 9-G, or NL4-3 sequences. The RRE sequences used are shown in Fig. 1B and the plasmid numbers of the RRE-containing constructs are listed in Table S3.

To create stocks of pseudotyped, packaged assay constructs, 293 T/17 cells were plated at a density of 3 × 10^6^ cells per 10 cm plate in IMDM supplemented with 10% BCS and gentamicin. One day after plating, the cell medium was replaced with IMDM supplemented with 5% BCS. The cells were then transfected with 15 ug of the desired Rev- or RRE-containing construct, 2.54 ug of the VSV-G expression plasmid pMD2.G (a gift from Didier Trono, Addgene plasmid # 12259), and 6.42 ug of the appropriate helper construct (pHit-CMV-GagPol^56^ for Rev-containing constructs and psPAX2 for RRE-containing constructs) using the PEI method^50^. The plasmid psPAX2 was a gift from Didier Trono (Addgene plasmid # 12260). Medium was collected from the producer culture 48 h after transfection, aliquoted, and frozen at −80 °C until use.

Transduction for vector titering and functional assays was performed in 96 well plates. A total of 2.5 × 10^5^ SupT1 cells suspended in PBS were seeded in each well along with stocks of vectors. Sterile DEAE-dextran was added to each well to a final concentration of 8 mcg/mL. The transduction cultures were then centrifuged at 380 RCF for 1 h at room temperature. After centrifugation, vector-containing medium was removed from the cell pellets, and cells were resuspended in SupT1 growth medium. Seventy-two hours after transduction, cells were again centrifuged briefly to pellet them, and the medium was removed. Cells were resuspended in cold PBS and held on ice in preparation for flow cytometry.

Transductions for determining the titer of vector stocks were performed using serial 1:10 dilutions of the initial stock. Transductions for the functional assays were performed using both Rev- and RRE-containing vectors at a target MOI of 0.18. Transductions for each Rev-RRE pair included in an experimental run were performed in duplicate or triplicate in different wells.

Flow cytometry was performed using an Attune NxT flow cytometer with autosampler attachment (Thermo Fischer Scientific). Data was acquired using the Attune NxT software package.

Color compensation and data analysis was performed after acquisition using FlowJo v10.6.1 (FlowJo, LLC). Gates were constructed to identify single SupT1 cells. Using an untransduced SupT1 population, dependent gates were then constructed to define eBFP2 and mCherry positive populations.

For data analysis, only single cells successfully transduced with both a Rev-containing and RRE-containing vector were considered. For this population, the arithmetic mean fluorescent intensity of eGFP and eBFP2 was determined and the ratio of eGFP/eBFP2 was calculated as the measure of relative Rev-RRE functional activity. This calculation was performed for each well in each experimental run.

### Statistical analysis of functional assays

For each experimental run, the ratio of eGFP/BFP2 was calculated for each of the two or three wells transduced with a particular Rev-RRE pair. The activity measurement of each well corresponding to a Rev-RRE pair was averaged to represent a single estimate of Rev-RRE activity for that pair. Repeated measurements of Rev-RRE pair activity performed in different experimental runs on different days were considered to constitute replicates for the purposes of statistical analysis.

Wells in which less than 500 cells were co-transduced with both the Rev-containing and RRE-containing construct were deemed uninformative and not included in activity calculations. Wells in which either the Rev-containing or RRE-containing constructs transduced more than 31% of cells were also excluded from further calculations, as a significant number of cells in these wells would contain two or more integrations of the Rev or RRE construct.

While the relative activity of Rev-RRE pairs in comparison to each other was consistent across experimental runs, the absolute ratio of mean eGFP/eBFP2 signal varied from transduction to transduction. Relative Rev activity on a particular RRE was compared using a linear mixed model by restricted maximum likelihood, with individual experimental run as a random effect and Rev as a fixed effect. Analysis was performed using R v4.1.2 and the lme4^57^ and lmerTest packages^58^. The *p* value was adjusted for multiple comparisons using the Hochberg method^59^. Activity measurements for any Rev-RRE pair represent at least three replicated experimental runs. The estimated relative activity and 95% confidence interval for each Rev-RRE pair was calculated from all available experimental runs. This single set of values is used in each figure above where relevant to compare the activity of Rev-RRE pairs. The activity of each Rev-RRE pair was normalized such that the activity of the 8-G Rev/NL4-3 RRE pair is has the value of 1 in each figure.

### Structural analysis of 8-G and 9-G Rev

Predicted structures of 8-G and 9-G Rev were calculated using the AlphaFold Collab utility (https://colab.research.google.com/github/deepmind/alphafold/blob/main/notebooks/AlphaFold.ipynb) with default settings^32^. The computed structures were compared with a previously published crystal structure of HIV-1 NL4-3 Rev^33,34^ using PyMOL Molecular Graphics System, version 2.5.1 (Schrödinger, LLC).

## Supplementary Information

Supplementary Information.

## Data Availability

The datasets generated during the current study and plasmids used to perform assays are available from the corresponding author on reasonable request. Nucleotide sequences including *rev* and the RRE for viruses 8-G, 9-G, and NL4-3 are available from GenBank (accession FJ389367, JX140676, and U26942, respectively).
