# Supplementary material for: HIV-1 Rev-RRE functional activity in primary isolates is highly dependent on minimal context-dependent changes in Rev
Source: Sci Rep. 2022 Nov 1;12:18416. doi: 10.1038/s41598-022-21714-2 (PMC9626594; doi:10.1038/s41598-022-21714-2)
Supplement: Supplementary file 1 — Supplementary Information. [file 41598_2022_21714_MOESM1_ESM.pdf]

Supplemental Material.

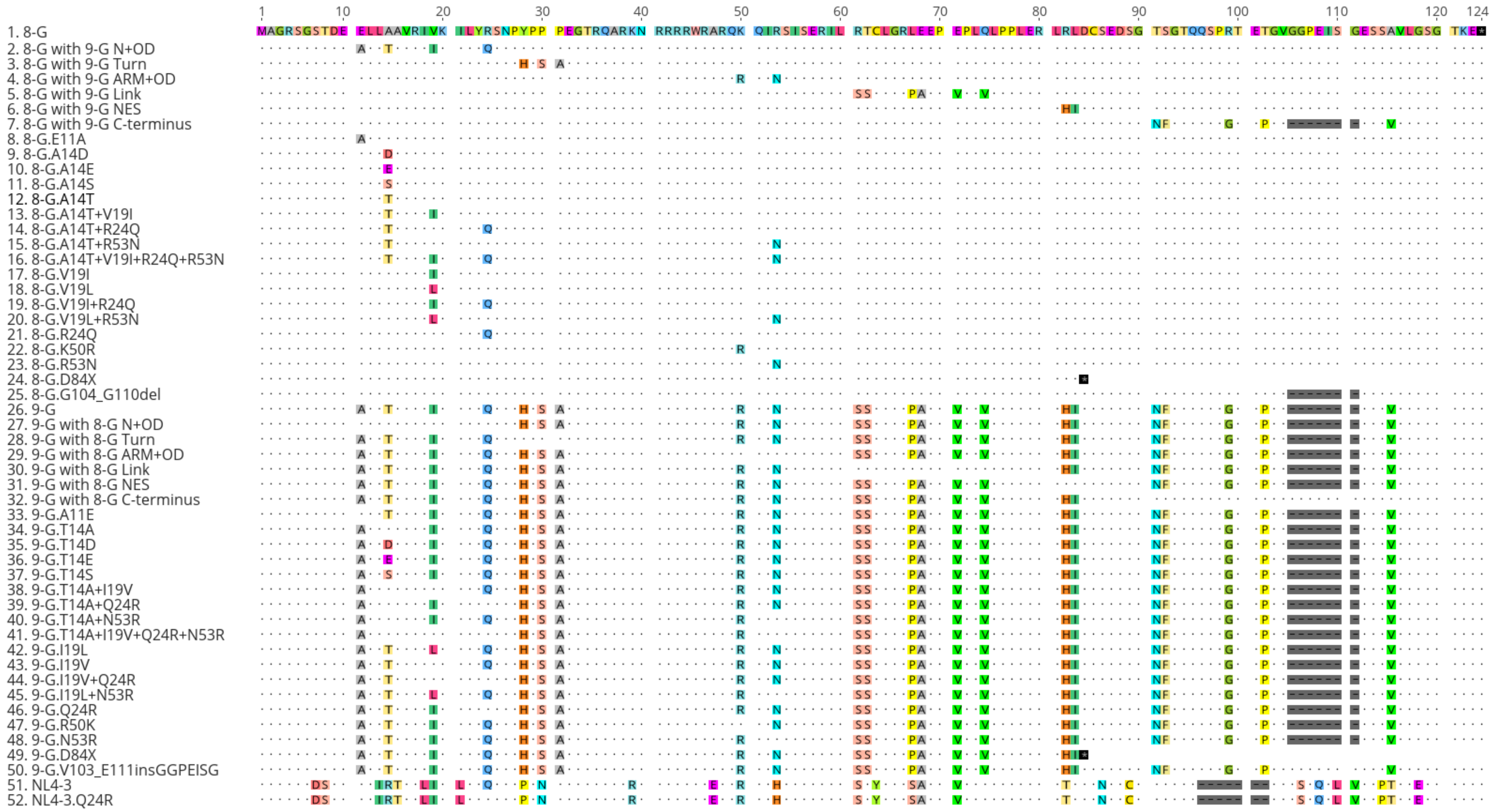

Figure S1. Alignment of Rev amino acid sequences.

The amino acid sequence of each Rev construct used in the functional assays was aligned. The sequence of the native 8-G Rev is used as the reference. Dots represent no change from the reference sequence. Dashes represent a deletion relative to the reference. * represents a stop codon. Sequences are numbered to facilitate referencing with the text.

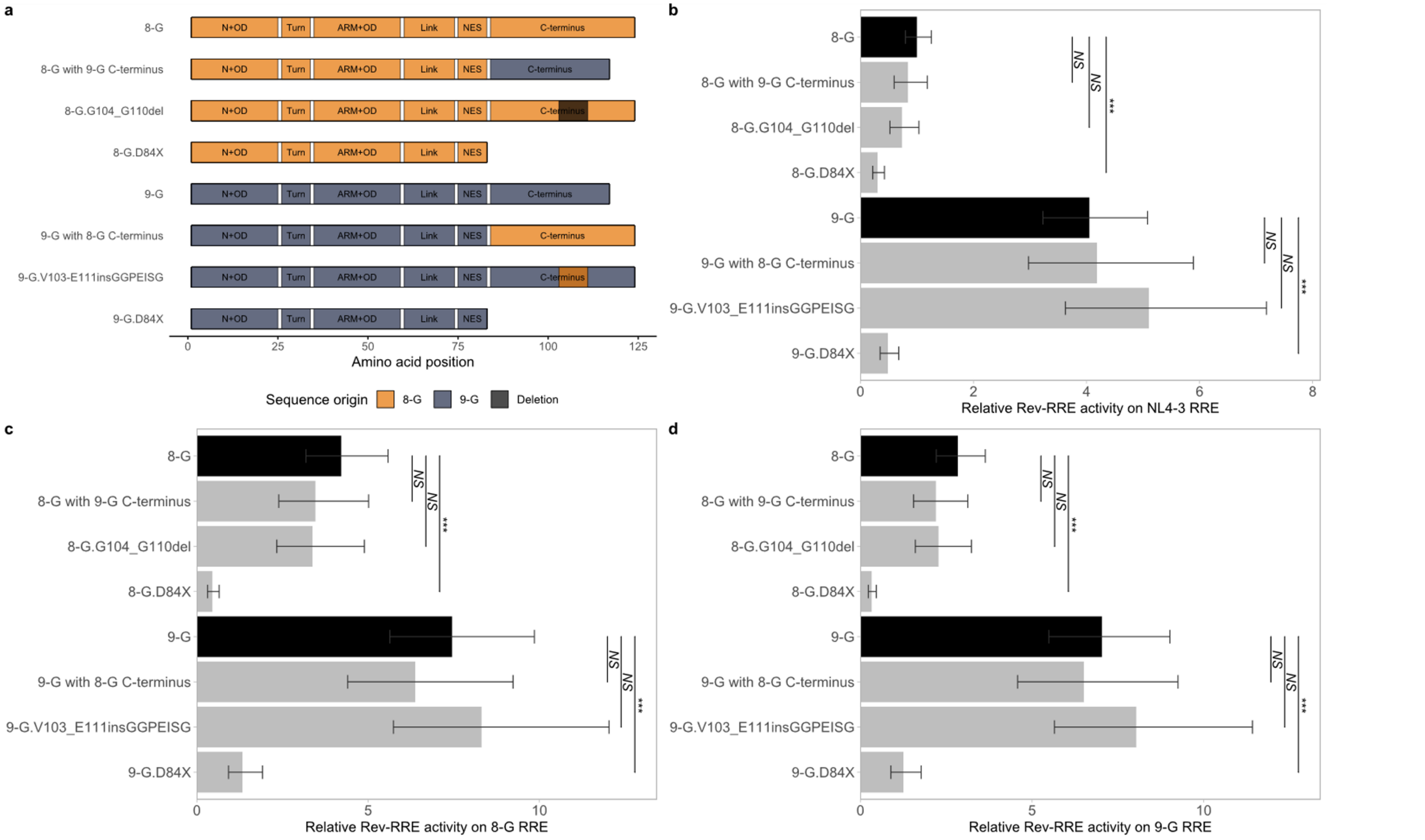

Figure S2. Activity of Revs with C-terminus modifications.

8-G and 9-G Revs were created with modifications to the C-terminal region and their functional activity was determined using the fluorescence-based assay system. A schematic of each Rev construct used in this set of experiments is shown in (a). In plots b-d, the native 8-G and 9-G Rev sequences are included in dark bars for reference and modified Revs are in light bars. The Revs were tested with the NL4-3 RRE (b), 8-G RRE (c), and 9-G RRE (d). N+OD – N-terminus plus first portion of the oligomerization domain, ARM+OD – arginine rich motif plus second portion of the oligomerization domain, NES – nuclear export signal. Relative activity is expressed in arbitrary units with the activity of the 8-G Rev/NL4-3 RRE pair defined as 1. $N \geq 3$ for all data points, error bars represent 95% CI. *NS* – not significant, *** $p < 0.001$.

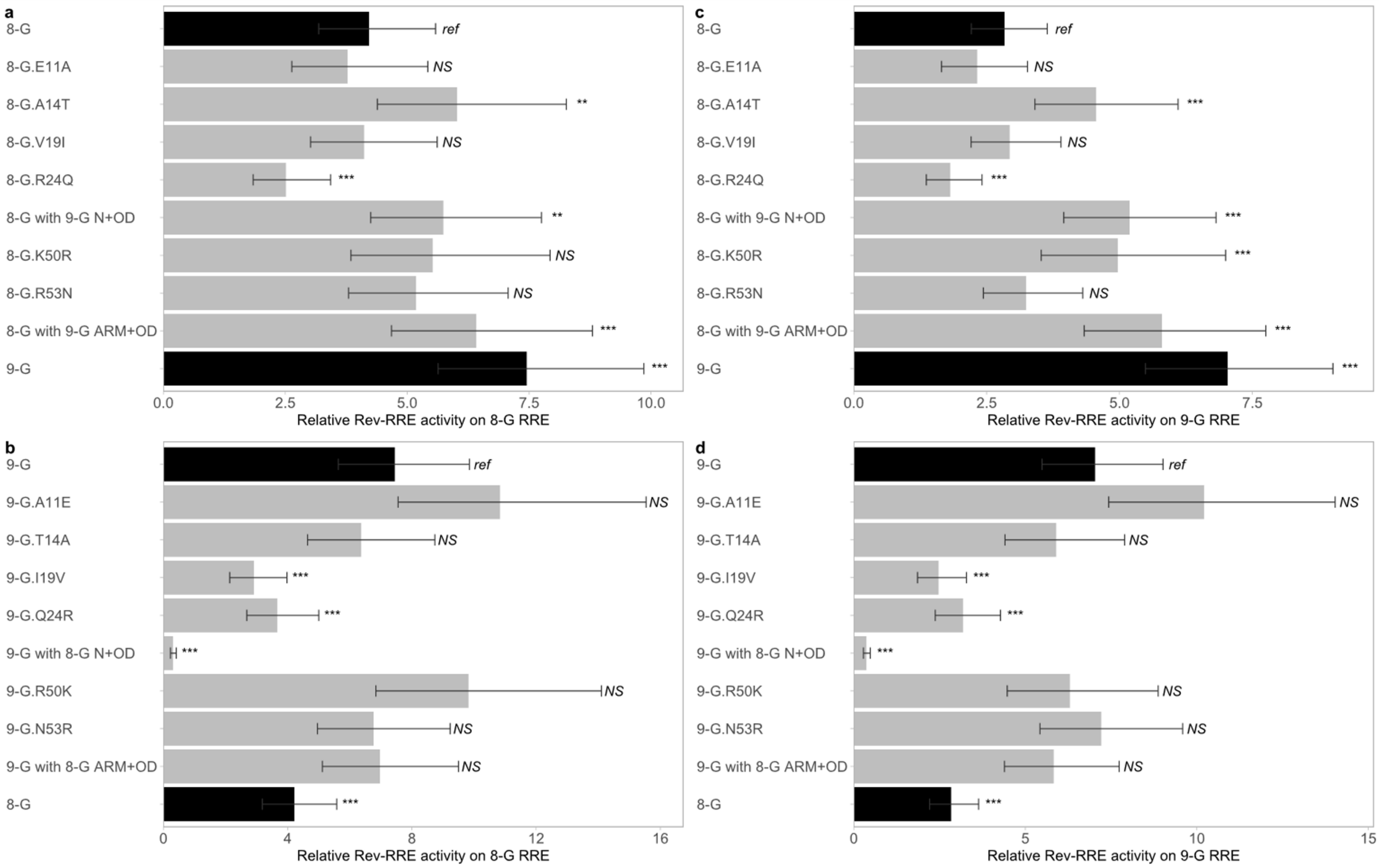

Figure S3. Activity of Revs with single amino acid substitutions on 8-G and 9-G RREs.

Revs with single amino acid substitutions within the N-terminal, OD, or ARM regions were created and their functional activity determined using the fluorescence-based assay system. a. 8-G derived Revs tested on the 8-G RRE. b. 9-G derived Revs tested on the 8-G RRE. c. 8-G derived Revs tested on the 9-G RRE. d. 9-G derived Revs tested on the 9-G RRE. In each plot, the native 8-G and 9-G Rev sequences are included in dark bars for reference and modified Revs are in light bars. For all plots, relative activity is expressed in arbitrary units with the activity of the 8-G Rev/NL4-3 RRE pair defined as 1. The statistical comparison is performed in reference to the top-most Rev in each plot. $N \geq 3$ for all data points, error bars represent 95% CI. *Ref* – reference sequence for the plot, *NS* – not significant, *** $p < 0.001$, ** $p < 0.01$.

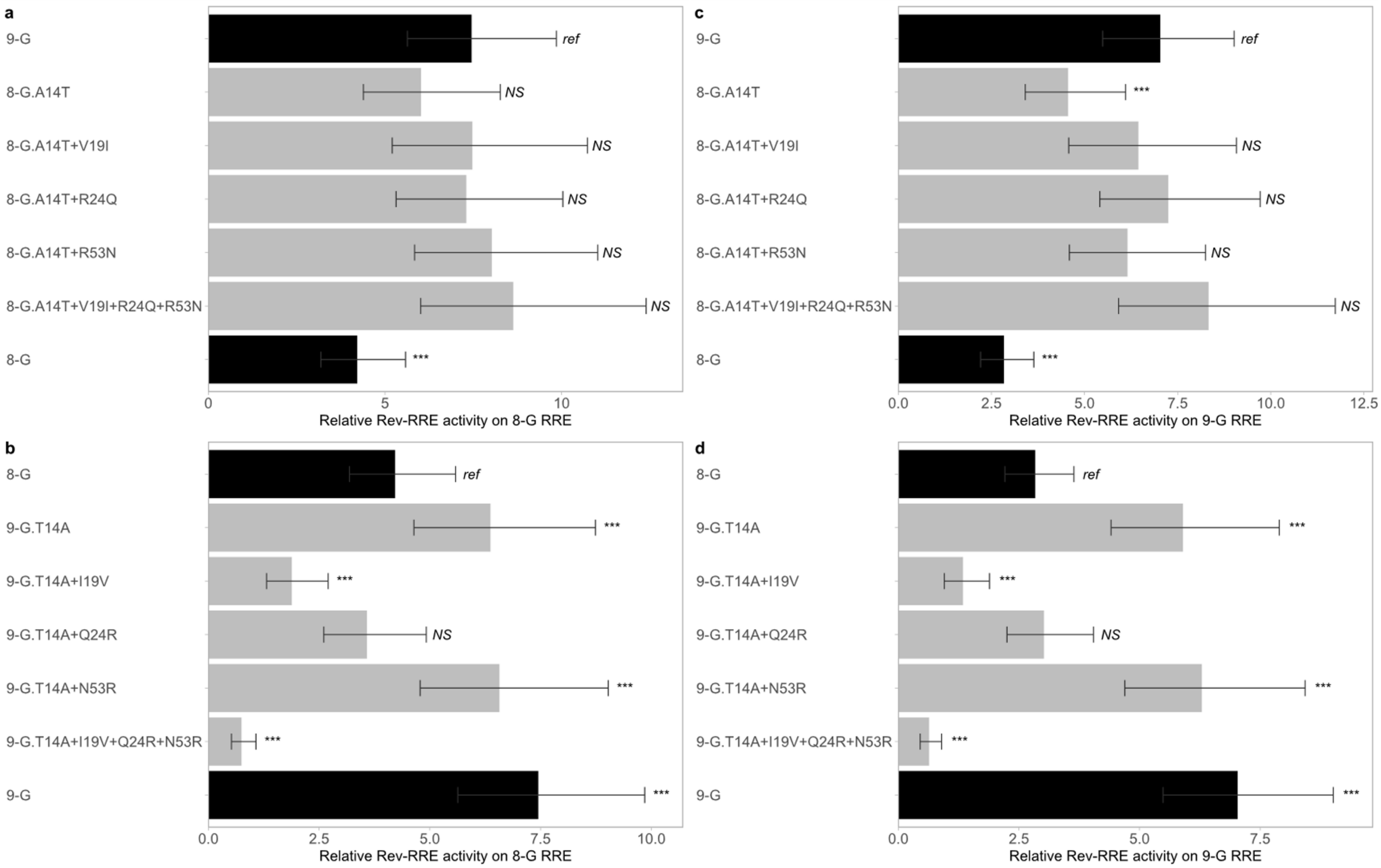

Figure S4. Activity of Revs with multiple amino acid substitutions on 8-G and 9-G RREs.

Revs with multiple amino acid substitutions were created and their functional activity determined using the fluorescence-based assay system. a. 8-G derived Revs tested on the 8-G RRE. b. 9-G derived Revs tested on the 8-G RRE. c. 8-G derived Revs tested on the 9-G RRE. d. 9-G derived Revs tested on the 9-G RRE. In each plot, the native 8-G and 9-G Rev sequences are included in dark bars for reference and modified Revs are in light bars. For all ploys, relative activity is expressed in arbitrary units with the activity of the 8-G Rev/NL4-3 RRE pair defined as 1. The statistical comparison is performed in reference to the top-most Rev in each plot. $N \geq 3$ for all data points, error bars represent 95% CI. *Ref* – reference sequence for the plot, *NS* – not significant, *** $p < 0.001$.

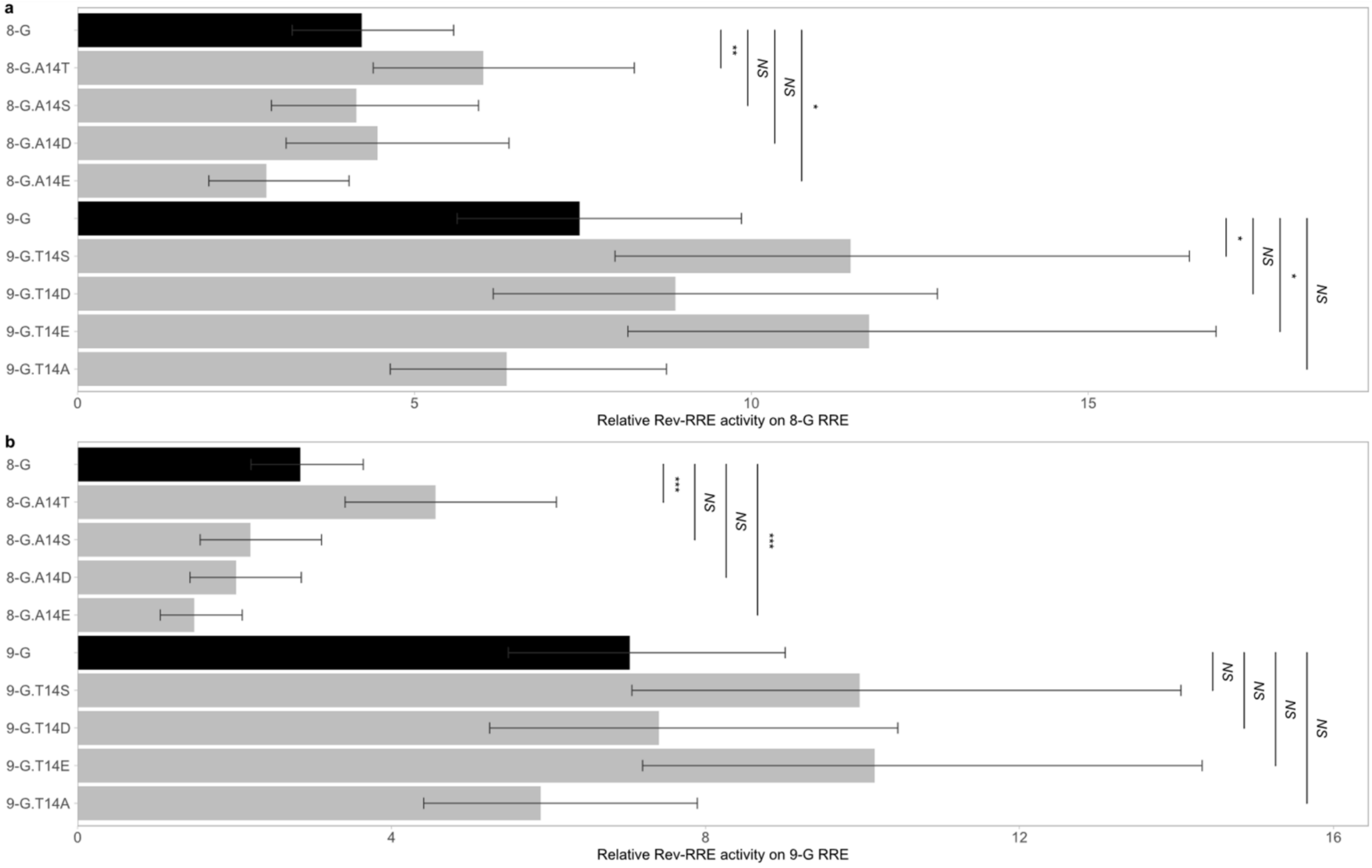

Figure S5. Activity of Revs with phosphomimetic substitutions on 8-G and 9-G RREs.

Revs with phosphomimetic amino acid substitutions at position 14 were created and their functional activity determined on the 8-G RRE (a) and 9-G RRE (b) using the fluorescence-based assay system. The native 8-G and 9-G Rev sequences are included in dark bars for reference and modified Revs are in light bars. For both plots, relative activity is expressed in arbitrary units with the activity of the 8-G Rev/NL4-3 RRE pair defined as 1. The statistical comparison is performed is performed between the Revs indicated by the vertical bars. $N \geq 3$ for all data points, error bars represent 95% CI. *NS* – not significant, *** $p < 0.001$, ** $p < 0.01$, * $p < 0.05$.

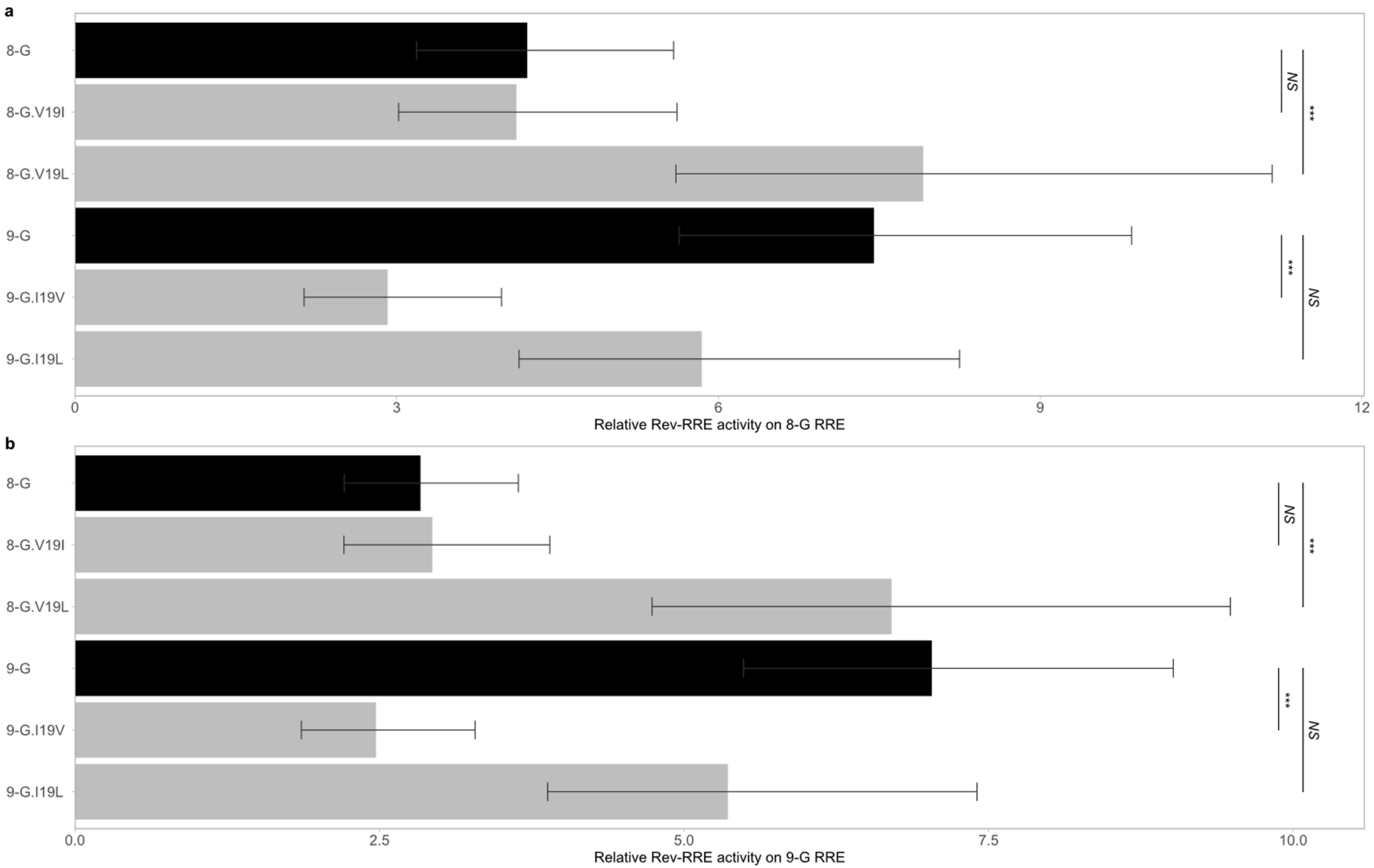

Figure S6. Activity of position 19 mutants on 8-G and 9-G RREs.

Revs with amino acid substitutions at position 19 were created and their functional activity determined on the 8-G RRE (a) and 9-G RRE (b) using the fluorescence-based assay system. The native 8-G and 9-G Rev sequences are included in dark bars for reference and modified Revs are in light bars. For both plots, relative activity is expressed in arbitrary units with the activity of the 8-G Rev/NL4-3 RRE pair defined as 1. The statistical comparison is performed is performed between the Revs indicated by the vertical bars. $N \geq 3$ for all data points, error bars represent 95% CI. *NS* – not significant, *** $p < 0.001$.

Table S1. List of replication-competent constructs.

Construct	Plasmid number
pNL4-3(Rev-)(8-G Rev-IRES-Nef)	5830
pNL4-3(Rev-)(9-G Rev-IRES-Nef)	5831

Table S2. List of Rev assay constructs.

Rev construct	Plasmid number
NL4-3	5864
9-G	5866
8-G	5868
8-G with 9-G Link	5912
8-G with 9-G Turn	5914
8-G with 9-G C-terminus	5916
8-G.R24Q	5918
9-G with 8-G Link	5920
9-G with 8-G Turn	5922
9-G with 8-G C-terminus	5924
9-G.Q24R	5926
8-G.G104_G110del	5928
9-G.V103_E111insGGPEISG	5930
8-G with 9-G ARM+OD	5982
8-G with 9-G N+OD	5984
8-G with 9-G NES	5986
9-G with 8-G ARM+OD	5988
9-G with 8-G N+OD	5990
9-G with 8-G NES	5992
NL4-3.Q24R	5994
8-G.A14T	6080
8-G.D84X	6081
8-G.E11A	6082
8-G.V19I+R24Q	6083
8-G.V19I	6084
9-G.A11E	6085
9-G.D84X	6086
9-G.I19V+Q24R	6087
9-G.I19V	6088
9-G.T14A	6089
8-G.A14T+R24Q	6105
8-G.A14T+R53N	6106
8-G.A14T+V19I	6107
8-G.A14T+V19I+R24Q+R53N	6108
8-G.K50R	6109
8-G.R53N	6110
9-G.N53R	6111
9-G.R50K	6112

9-G.T14A+I19V	6113
9-G.T14A+I19V+Q24R+N53R	6114
9-G.T14A+N53R	6115
9-G.T14A+Q24R	6116
8-G.A14D	6406
8-G.A14E	6407
8-G.A14S	6408
9-G.T14D	6409
9-G.T14E	6410
9-G.T14S	6411
9-G.I19L+N53R	6666
9-G.I19L	6667
8-G.V19L+R53N	6668
8-G.V19L	6669

Table S3. List of RRE assay constructs.

**RRE construct**	**Plasmid number**
NL4-3	5936
8-G	5938
9-G	5940
